# SAGES guidelines for the management of comorbidities relevant to metabolic and bariatric surgery

**DOI:** 10.1007/s00464-024-11433-2

**Published:** 2024-12-11

**Authors:** Sunjay S. Kumar, Claire Wunker, Amelia Collings, Varun Bansal, Theofano Zoumpou, Julietta Chang, Noe Rodriguez, Andrew Sabour, Lisa Renee Hilton, Omar M. Ghanem, Bradley S. Kushner, Lindsey Jean Loss, Essa M. Aleassa, Ivy N. Haskins, Subhashini Ayloo, Adam Reid, David Wayne Overby, Peter Hallowell, Tammy Lyn Kindel, Bethany J. Slater, Francesco Palazzo

**Affiliations:** 1 Department of Surgery, Thomas Jefferson University Hospital, Philadelphia, PA USA; 2 Department of Surgery, Saint Louis University, St. Louis, MO USA; 3 Department of Surgery, University of Louisville, Louisville, KY USA; 4 Department of Surgery, University of Colorado, Aurora, CO USA; 5 Department of Surgery Rutgers, New Jersey Medical School, Newark, NJ USA; 6 Department of Surgery, Kaiser Permanente Bellevue Medical Center, Baldwin Park, CA USA; 7 Department of Surgery, Cleveland Clinic, Cleveland, OH USA; 8 Department of Surgery, University of Nevada, Las Vegas, NV USA; 9 Department of Surgery, Medical College of Georgia, Augusta, Georgia; 10 Department of Surgery, Mayo Clinic, Rochester, MN USA; 11 Department of Surgery, Washington University in St. Louis, St. Louis, MO USA; 12 Department of Surgery, Oregon Health and Science University, Portland, OR USA; 13 Department of Surgery, University of Nebraska Medical Center, Omaha, NE USA; 14 Department of Surgery, Brown University, Providence, RI USA; 15 Department of Surgery, Southern Illinois University School of Medicine, Springfield, IL USA; 16 Department of Surgery, University of North Carolina, Chapel Hill, NC USA; 17 Department of Surgery, University of Virginia, Charlottesville, VA USA; 18 Department of Surgery, Medical College of Wisconsin, Madison, WI USA; 19 Department of Surgery, University of Chicago, Chicago, IL USA

**Keywords:** Intraoperative cholangiography, Choledocholithiasis, Inflammatory bowel disease, Gastroesophageal reflux disease

## Abstract

**Background:**

Patients who are under consideration for or have undergone metabolic and bariatric surgery frequently have comorbid medical conditions that may make their perioperative care more complex. These recommendations address routine intraoperative cholangiography in patients with bypass-type anatomy, the management of reflux disease after sleeve gastrectomy, and the optimal bariatric procedure for patients with comorbid inflammatory bowel disease.

**Methods:**

A systematic review was conducted including studies published from 1990 to 2022 to address these questions. These results were then presented to a panel of bariatric surgeons who formulated recommendations based on the best available evidence or utilized expert opinion when the evidence base was lacking.

**Results:**

Conditional recommendations were made in favor of routine intraoperative cholangiography in patients with bypass-type anatomy undergoing laparoscopic cholecystectomy, trialing medical management prior to surgical management in patients with reflux after sleeve gastrectomy, and sleeve gastrectomy rather than Roux en Y gastric bypass in patients with inflammatory bowel disease. The strength of these recommendations was limited by the quality of evidence available. Recommendations for future research were made for all questions.

**Conclusions:**

These recommendations should provide guidance regarding management of these comorbidities in patients who are under consideration for or have undergone metabolic and bariatric surgery. These recommendations also identify important areas where the future research should focus to strengthen the evidence base.

**Supplementary Information:**

The online version contains supplementary material available at 10.1007/s00464-024-11433-2.

## Executive summary

### Background

As the obesity epidemic continues to worsen, more patients are seeking metabolic and bariatric surgery. These patients frequently have comorbid conditions that can affect their perioperative care. A panel of bariatric surgeons was assembled to provide recommendations regarding routine intraoperative cholangiography in patients with bypass-type anatomy, the management of reflux disease after sleeve gastrectomy, and the optimal bariatric procedure for patients with comorbid inflammatory bowel disease.

### Methods

Systematic literature reviews were conducted for three key questions regarding comorbidities in patients undergoing metabolic and bariatric surgery. Questions were chosen by members of the SAGES Guidelines Committee who practice metabolic and bariatric surgery with guidance from senior members of the Guidelines Committee. The Cochrane Library, Clinicaltrials.gov, Embase, PubMed, and the International Clinical Trials Registry Platform were searched to identify randomized controlled trials and non-randomized comparative studies. Evidence-based recommendations were formulated using the Grading of Recommendations Assessment, Development, and Evaluation (GRADE) methodology by subject matter experts. GRADE is a transparent framework used in the development of clinical practice guidelines, using the highest-level evidence available. Expert opinion was utilized in cases of insufficient data for an evidence-based recommendation. During the manuscript revision process, an experienced patient representative from the Obesity Action Network reviewed the importance ascribed to the outcomes by the panel and the final recommendations themselves. Recommendations for future research were also proposed.

### Interpretation of strong and conditional recommendations

All recommendations were assigned either a “strong” or “conditional” recommendation. The words “the guideline panel recommends” are used for strong recommendations, and “the guideline panel suggests” for conditional recommendations, as per the GRADE approach. A conditional recommendation signals that the benefits of adhering to a recommendation probably outweigh the harms although it does also indicate uncertainty. This uncertainty may be due to a lack of high-quality evidence or variability in how individual patients value the outcomes of interest.

### How to use this document

The primary aim of these recommendations is to provide guidance for the management of comorbid conditions in patients who are under consideration for or who have undergone metabolic and bariatric surgery. They are also intended to provide education for patients, inform advocacy, and describe future areas for research. Given the lack of strong evidence, these recommendations provide guidance but not mandates. The wide variety of clinical scenarios will require adjustment of treatment plans to suit the needs and priorities of the individual patient. Finally, because these recommendations take a patient-centered approach as opposed to a health systems-centered approach, patients can use these recommendations as a source of information and basis for discussion with their physicians.

### Recommendations

#### KQ1. Should routine IOC or alternative options be used for patients with gastrointestinal bypass-type anatomy (RYGB and DS, etc.) undergoing cholecystectomy?

The panel suggests routine intraoperative imaging of the biliary anatomy in patients with bypass-type anatomy undergoing laparoscopic cholecystectomy (expert opinion recommendation due to low-quality of evidence).

#### KQ2. Should surgical or medical therapy be used for GERD post-sleeve gastrectomy?

The panel suggests medical therapy be trialed as first line therapy in patients with GERD post-sleeve gastrectomy (expert opinion recommendation due to low-quality of evidence).

If the patient has medically refractory GERD post-sleeve gastrectomy and has a BMI > 35, the panel suggests conversion to RYGB. For patients with medically refractory GERD with BMI < 35, the data is are clear on whether RYGB or magnetic sphincter augmentation (MSA) would be superior. For patients with a BMI between 30 and 35, consideration should be given for gastric bypass if the patient has associated metabolic diseases. For patients with BMI < 30, potential options for antireflux surgery include MSA and procedures that include gastric diversion.

#### KQ3. Should sleeve gastrectomy or Roux en Y gastric bypass be used for obese patients with IBD undergoing metabolic and bariatric surgery?

The panel suggests sleeve gastrectomy rather than bypass for obese patients with IBD (conditional recommendation, very low certainty evidence).

## Introduction

### Aim of these recommendations and specific objectives

The aim of these recommendations by the Society of American Gastrointestinal and Endoscopic Surgeons (SAGES) is to provide recommendations regarding decision-making for patients with obesity and other conditions such as biliary disease, gastroesophageal reflux disease (GERD), or Inflammatory Bowel Disease (IBD). Specifically, this manuscript seeks to provide recommendations regarding: a) performing a routine intraoperative cholangiogram (IOC) during cholecystectomy in patients with bypass-type anatomy; b) treating post-sleeve gastrectomy GERD medically or surgically; and c) whether one of the available bariatric operations is safer in patients with IBD, who represent a complex population. The panel chose topics that were felt to be impactful in the care of these patients that has not already been addressed in other society guidelines.

The target audience of these recommendations includes surgeons, gastrointestinal specialists, all physicians and allied health care providers who care for patients before and after metabolic and bariatric surgery, and the patients themselves. Policy makers and insurance providers involved in delivering health care services related to these topics or evaluating direct and indirect benefits, harms, and costs related to these procedures may also consider these recommendations in their deliberations.

### How to use these guidelines

The aim of these guidelines is to assist surgeons, gastroenterologists, metabolic disease specialists, and other physicians involved in the care of patients undergoing metabolic and bariatric surgery to make decisions about the management of certain disease processes. These guidelines are also intended to provide education, inform advocacy, and describe future areas for research. While these are meant to highlight the optimal approach in a generalized patient population, distinct patient needs, comorbidities, and specific situations may require adjustments to determine the ideal treatment for each individual. In addition, these guidelines can serve as a resource for patients to promote discussion with their physicians. Given the above, this guideline will be available without paywall at sages.org.

## Methods

### Guideline panel organization

An expert panel of practicing surgeons was selected from within the SAGES Guidelines committee to create the key questions for this metabolic and bariatric surgery guideline. Surgeons were chosen on the basis of their clinical work and academic interests. The systematic review was overseen by a methodologist with systematic review expertise (A.A.). The systematic review team included panel members and trainee members of the Guidelines Committee. A methodologist (M.T.A.) with guideline development expertise and the guideline committee fellow (S.S.K.) facilitated guideline panel meetings as non-voting members of the panel. The panel used the Grading of recommendations, assessment, development, and evaluations (GRADE) methodology to assess the evidence from the systematic review and judge certainty of evidence and the strength of guideline recommendation [1, 2]. Reporting of the guideline adheres to the *Essential Reporting Items for Practice Guidelines in Healthcare* (RIGHT) checklist [3].When the evidence was found to be too weak for an evidence-based recommendation, the panel utilized expert opinion instead. Full author roles are listed in Appendix A.

### Guideline funding & declaration and management of competing interests

SAGES provided funding for the librarian who assisted with the systematic review, the methodologists, and the Guidelines Committee Fellow. Industry did not provide any input whatsoever into the conception or development of this guideline. A standard SAGES conflict of interest form was collected from each coauthor by the lead (F.P.). There were no conflicts of interest identified. A full list of declarations is listed in Appendix B.

### Selection of questions and outcomes of interest

The guideline panelists developed key questions (KQs) relevant to metabolic and bariatric surgery according to the Population, Intervention, Comparison, Outcome (PICO) format, in consultation with the methodologist, guideline lead (F.P.), and committee chair (B.S.).

The panel members used their clinical experience to identify patient-centered outcomes they believed most surgeon–patient dyads would consider important to decision-making. These outcomes were chosen based on panel consensus by simple majority and then further designated as critical or important to decision-making on the basis of their relative importance to patients. This designation was confirmed by panel members during the formulation of recommendations after reviewing the evidence from the systematic review.

### Evidence review and synthesis

A systematic review addressing the KQs was conducted according to the SAGES Guidelines Committee’s standard operating procedure [4]. The Cochrane Library, Clinicaltrials.gov, Embase, PubMed, and the International Clinical Trials Registry Platform were searched from 1990 to 2022. When no direct comparative studies were available, non-comparative evidence was utilized. Search strategies can be found in Appendix C.

Each record was screened by two independent reviewers at both the abstract and full text levels. Screening criteria and the Preferred Reporting Items for Systematic Reviews and Meta-Analyses (PRISMA) diagrams can be found in Appendix D. As there was no RCT data found for any question, study quality was assessed using the Newcastle Ottawa scale. Random effects meta-analysis was performed on the extracted data [5]. Forest plots can be found in Appendix E.

### Determining the certainty of evidence

As per the Guidelines Committee’s standard operating procedure, the GRADE approach was utilized to judge the certainty of evidence available for each outcome [1, 6]. The highest-level of evidence identified was imported into GRADEPro evidence tables [7]. The certainty of this evidence was evaluated on the basis of its risk of bias, inconsistency, indirectness, and imprecision. The certainty was downgraded based on the number of domains across which there were concerns. These data were then imported into an Evidence to Decision table for each KQ which provided the framework through which the expert panel developed its recommendations. Evidence tables and Evidence to Decision tables can be found in Appendices F and G, respectively.

### Assumed values and preferences

The panel members used clinical experience to inform judgment on the valuation of different outcomes on behalf of patients. This expertise was deemed likely sufficient to anticipate the variation in values by patients informed by the same evidence. Empiric evidence of how patients value these outcomes was not searched, so the panel’s judgment was used as a proxy for patient values and preferences. As described below, these judgements were evaluated by an experienced patient representative during the review process.

### Development of recommendations

The panel convened virtually in the summer of 2023 to review the evidence and make recommendations. The results of the systematic review and the articles utilized were available for independent review prior to the meetings. During the meetings, the panel members reviewed the evidence and completed the Evidence to Decision tables to generate recommendations. This process entailed deliberating the magnitude of desirable and undesirable effects, the certainty of evidence, and variation in how patients may value outcomes. After this, the panel voted on whether the overall balance of these considerations favored the intervention or comparison. The panel then discussed the acceptability and feasibility of this judgment. For each decision, both the available evidence was discussed as well as pertinent additional considerations taken either from panel expert experience or interpretation of evidence. Based on the balance of effects and the acceptability and feasibility of a favored option, the panel voted on the final recommendation for that key question. While serial voting was used to come to a consensus on individual components of the EtD, 80% agreement was mandatory for all final recommendations.

Subgroups were discussed in the justification for each recommendation and are specified for each KQ where relevant. Full evidence to decision tables are presented in Appendix G and summarized in the following recommendations.

### Guideline document review

This guideline was drafted based on the evidence to decision tables and panel discussion and was edited by all panel members. During the manuscript revision process, an experienced patient representative from the Obesity Action Network reviewed the importance ascribed to the outcomes by the panel and the final recommendations themselves. In accordance with SAGES Guidelines Committee policies, the manuscript was then submitted to SAGES Executive board for approval and published online for public comment for 4 weeks.

### Recommendation for future research

The authors have provided a concise and comprehensive review of potential avenues for future research for each KQ discussed. These were made based on existing gaps in the literature identified during the review process. These recommendations are listed at the end of each KQ.

## Key questions

KQ1: Should routine IOC or alternative options be used for patients with gastrointestinal bypass-type anatomy (RYGB and DS, etc.) undergoing cholecystectomy?
KQ2: Should surgical or medical therapy be used for GERD post-sleeve gastrectomy?
KQ3: Should sleeve gastrectomy or Roux en Y gastric bypass be used for obese patients with IBD undergoing metabolic and bariatric surgery?

## Recommendations

### KQ1. Should routine IOC or alternative options be used for patients with gastrointestinal bypass-type anatomy (RYGB and DS, etc.) undergoing cholecystectomy?

The panel suggests routine intraoperative imaging of the biliary anatomy in patients with bypass-type anatomy undergoing laparoscopic cholecystectomy (expert opinion recommendation due to low-quality of evidence).

#### Introduction

Patients who have undergone metabolic and bariatric surgery are at increased risk of developing symptomatic gallstone disease [8–13]. Bypass-type operations alter the anatomy in a way that typically precludes standard endoscopic retrograde cholangiopancreatography (ERCP) as a viable treatment option. Such patients may require operative bile duct exploration, laparoscopic-assisted ERCP, or an Endoscopic Ultrasound Directed transGastric ERCP (EDGE). Because of the difficulties this situation poses, the panel sought to investigate whether IOC should be performed routinely when completing a cholecystectomy in a patient with bypass-type anatomy.

#### Summary of the evidence

Both elective and urgent laparoscopic cholecystectomies were included in this question. The literature search for this question revealed only single arm data. The panel deemed this insufficient for an evidence-based recommendation given the lack of direct, comparative data.

#### Decision criteria and additional considerations

The panel’s discussion focused on the potential difficulty of ERCP if a patient develops choledocholithiasis after a bypass-type operation. Traditional contrast-based cholangiography would be the panel’s first choice. However, given the proliferation of intraoperative biliary imaging techniques, if intraoperative cholangiography cannot be performed due to access or experience, the panel encouraged the use of other techniques, such as minimally invasive ultrasound or fluorescence-based cholangiography.

The panel discussed the routine versus selective use of cholangiography based on the patient’s history, laboratory studies, and imaging findings. The consensus was to suggest the use of routine cholangiogram when gastric bypass anatomy is present based on the unique opportunity to complete a transcystic exploration at the time of cholecystectomy should choledocholithiasis be confirmed.

Surgeons performing these studies should have the ability to intervene on biliary stones identified during imaging. This may involve simple flushing of the cholangiography catheter with the use of glucagon, endoscopic interventions utilizing SpyGlass-type technology, formal common bile duct (CBD) exploration, or even on-table ERCP in collaboration with a gastroenterologist.

This conditional recommendation would be stronger in patients with biochemical or imaging findings suggestive of CBD stones preoperatively. Similarly, in patient with a preoperative magnetic resonance cholangiopancreatography that demonstrates no evidence of CBD stones, it would be reasonable to omit performing IOC.

#### Patient representative commentary

The patient representative noted long-term adverse outcomes will generally be the most important to patients; as long as the short-term risks are reasonable and described in advance of the intervention, many patients would accept these to decrease the risk of future and potentially greater adverse events. Patients with choledocholithiasis who later need to undergo multiple ERCPs or operative intervention will also suffer greater financial costs, which argues in favor of performing IOC at the time of cholecystectomy. Lastly he noted that future studies should investigate the experience of patients suffering with these problems and their sequelae to better understand the emotional burden to patients.

#### Conclusions and research needs

There is a clear need for direct, comparative evidence investigating this question. Patients with bypass-type anatomy should be followed long-term to understand the rate of symptomatic CBD stones after laparoscopic cholecystectomy and whether intraoperative imaging at the time of cholecystectomy decreases this rate.

### KQ2. Should surgical or medical therapy be used for GERD post-sleeve gastrectomy?

The panel suggests medical therapy be trialed as first line therapy in patients with GERD post-sleeve gastrectomy (expert opinion recommendation due to low-quality of evidence).

If the patient has medically refractory GERD post-sleeve gastrectomy and has a BMI > 35, the panel suggests conversion to RYGB. For patients with medically refractory GERD with BMI < 35, the data is are clear on whether RYGB or MSA would be superior.

For patients with a BMI between 30 and 35, consideration should be given for gastric bypass if the patient has associated metabolic diseases. For patients with BMI < 30, potential options for antireflux surgery include MSA and procedures that include gastric diversion.

When present, a hiatal hernia should be surgically repaired and in select circumstances it may be the only surgical intervention required.

#### Introduction

GERD after sleeve gastrectomy is an incompletely understood phenomenon but multiple studies provide evidence for its existence [14]. Despite extensive preoperative discussion, evaluation, and counseling, pre-existing GERD may worsen or de novo GERD may develop after sleeve gastrectomy, requiring further interventions. Whether these patients derive greater benefit from medical management alone or surgical treatment remains under investigation.

#### Summary of the evidence

The literature identified three studies which contained low-quality, direct, comparative evidence for some of the outcomes which the panel had deemed critical to decision-making. Given the poor quality of evidence, the panel decided to make a recommendation based on expert opinion rather than the evidence alone.

#### Decision criteria and additional considerations

This is not truly an “either, or” question. Nearly every patient with GERD following sleeve gastrectomy will trial medical therapy prior to discussing surgical revision specifically for GERD, which the panel feels is completely appropriate.

The question becomes less clear when patients have medically refractory GERD. If surgical intervention is being considered and a hiatal hernia is present, this should be addressed at the time of surgery for the clear benefit of re-establishing normal distal esophageal anatomy. For patients with insufficient treatment response post-sleeve and BMI > 35, conversion to a malabsorptive bariatric procedure seems the most reasonable approach to address both problems with one operation. Similarly, such patients who also have a hiatal hernia should undergo repair of the hiatal hernia at the time of conversion to bypass-type anatomy.

For medically refractory patients with a BMI between 30 and 35, MSA and conversion to gastric bypass may both be reasonable options depending on individual patient characteristics and values. Patients at this BMI range with associated metabolic diseases may derive greater benefit from conversion to gastric bypass. Similarly, patients of Asian descent at this BMI range may derive greater benefit from conversion to gastric bypass as there is evidence they tend to develop metabolic syndrome at a lower BMI threshold than other patients [15]. If the patient has a hiatal hernia that is thought to be causing GERD, hiatal hernia repair alone may be a viable treatment option.

Patients with a BMI less than 30 may undergo MSA or gastric bypass as well. The potential for malnutrition in this patient population must be discussed extensively prior to proceeding with gastric bypass. As mentioned above, in selected patients with GERD and a hiatal hernia after sleeve gastrectomy, hiatal hernia repair alone may be sufficient.

All patients considering undergoing a second metabolic operation should be evaluated in a multidisciplinary fashion, just as they were prior to their first operation. The standard contraindications to gastric bypass still apply, including potential psychosocial issues that may place the patient at greater risk for malnutrition or nutrient deficiencies.

#### Patient representative commentary

The patient representative noted that this is an especially relevant and patient-centered topic as it is commonly discussed by patients suffering with GERD post-sleeve gastrectomy. While he did not feel the need to provide any specific critique of the way the panel discussed the problem, he again noted the importance of studying long-term sequelae of the various treatment options as well as the patient experience.

#### Conclusions and research needs

As described above, gastric bypass and MSA are both potential options for patients with medically refractory GERD post-sleeve gastrectomy but it is unclear at what BMI threshold most patients would derive more benefit from bypass than MSA. The patient’s individual values and preferences will also significantly impact this judgement.

Similarly, there should be further investigation into endoscopic therapies for these patients, such as anti-reflux mucosectomy.

### KQ3. Should sleeve gastrectomy or Roux en Y gastric bypass be used for obese patients with IBD undergoing metabolic and bariatric surgery?

The panel suggests sleeve gastrectomy rather than bypass for obese patients with IBD (conditional recommendation, very low certainty evidence).

#### Introduction

Approximately 30% of patients with IBD in the US have concomitant obesity [16, 17]. The principles of surgical management for IBD, which typically focus on organ preservation and conserving intestinal length, may be at odds with malabsorptive bariatric operations. The gastrojejunal and jejunojejunal anastomoses may also be at greater risk for complications in patients with chronic small bowel inflammation.

##### Summary of the evidence

A total of six observational cohort studies reporting on the outcomes deemed important by the panel were identified [18–23]. All six studies included a mixed cohort of patients with ulcerative colitis and Crohn’s disease, prior intestinal resections, and active medication use (Table 1).

**Table 1:** KQ3 inflammatory bowel disease study demographics

Study name	Patients	UC	CD	Unclassified	Prior resection	On IBD meds
Aelfers	45	16	29	0	9	27
Aminiam	20	13	7	0	10	11
Heshmati	54	23	31	0	8	30
Hudson	13	4	9	0	7	6
McKenna	31	20	10	1	11	9
Reenaers	85	20	64	1	19	69

The evidence base was too limited to draw distinctions between ulcerative colitis and Crohn’s disease as well as active and quiescent disease. Given this and the retrospective nature of these studies, the overall certainty of evidence was rated very low primarily due to baseline differences between the two cohorts as well as small sample sizes.

Perioperative complications were defined as Clavien Dindo grade 2 and greater.

There were no mortalities in the gastric bypass population within the studies with comparative evidence.

##### Benefits

There were five outcomes found to be benefits to utilizing sleeve gastrectomy rather than RYGB. Single arm data were used for the outcome of mortality as the direct comparative data had too few patients for any outcomes in the RYGB cohort, given that this is a relatively rare outcome.

Perioperative complications: 166 fewer per 1,000 patients (95% CI 214 fewer to 48 fewer) based on 5 observational studies with 191 patients.

Long term complications: 135 fewer per 1,000 patients (95% CI 169 fewer to 26 fewer) based on 4 observational studies with 159 patients.

IBD worsening: 182 fewer patients reporting worsening per 1,000 patients (95% CI 208 fewer to 11 more) based on 1 observational study with 54 patients: 51 fewer episodes of ulceration per 1000 patients (95% CI 59 fewer to 110 more) based on two observational studies with 49 patients: 49 fewer episodes of obstruction, hemorrhage, fistula, or perforation per 1,000 patients (95% CI 69 fewer to 79 more) based on three observational studies with 99 patients: 117 fewer episodes of pain requiring medical therapy per 1,000 patients (95% CI 132 fewer to 8 more) based on three observational studies with 83 patients.

Mortality: 13 fewer per 1,000 patients (95% CI 143 fewer to 76 more) based on 6 observational studies with 219 patients.

Reoperation: 126 fewer per 1,000 patients (95% CI 137 fewer to 80 fewer) based on 6 observational studies with 220 patients.

##### Harms and burden

There were no harms identified to utilizing sleeve gastrectomy rather than RYGB.

##### Decision criteria and additional considerations

The panel acknowledges that a colorectal surgeon was not included in this panel discussion.

Overall the panel found the balance of effects favored sleeve gastrectomy. The conditional recommendation may be stronger in patients who are immunosuppressed or who have undergone prior small bowel resections. However, in patients with very high BMIs, concomitant GERD, or quiescent IBD, greater consideration may be given to RYGB. Whether the patient has ulcerative colitis or Crohn’s disease is also likely to affect this decision. Patients with ulcerative colitis who undergo proctocolectomy may later be considered for an ileal pouch anal anastomosis, which is not a straightforward decision in patients with a RYGB. Extensive multidisciplinary evaluation is required in these patients.

##### Patient representative commentary

The patient representative agreed that there would likely be variability in how patients value the outcomes of interest based on how well-controlled their IBD is and whether they have ulcerative colitis or Crohn’s disease. He also agreed that patients with very high BMIs, GERD, or quiescent IBD may have a greater preference for RYGB.

##### Conclusions and research needs

Overall the panel found that the balance of effects based on limited evidence likely favored sleeve gastrectomy over RYGB. Important considerations that may modify this recommendation include immunosuppression, the nature of the patient’s IBD, the patient’s BMI, and concomitant GERD. These patients require extensive multidisciplinary discussion, including colorectal surgeons and gastroenterologists familiar with the natural history of these diseases, prior to undergoing any metabolic or bariatric surgical procedure.

Further research into IBD remission and recurrence in patients who have undergone bariatric operations is necessary to provide recommendations based on higher level evidence.

## Discussion

This guideline is the first clinical practice guideline from a major surgery society addressing IOC at the time of cholecystectomy in patients with bypass-type anatomy, the management of GERD after sleeve gastrectomy, and the choice of bariatric operation in patients with IBD.

The panel believes that it is feasible to successfully implement these recommendations into local practice. Their acceptance will be limited by the low-quality evidence on which they are based. However, the conditional nature of the recommendation allows surgeons to tailor their treatment approach to the individual patient without feeling they are in violation of this guideline.

Literature searches will be conducted every 3 years to identify new studies which could inform these recommendations. The results of the literature searches will be summarized by the Living Guidelines Committee and posted on the SAGES website accompanying these guidelines. If there is evidence contradicting the prior recommendations, the expert panel will be reconvened to provide updated recommendations.

The recommendations in this guideline are limited significantly by the quality of data available. This is discussed in more detail in both the summary of evidence and certainty of evidence for each KQ. Additional limitations include all participants being surgeons as opposed to also including gastroenterologists, metabolic disease specialists, or primary care physicians.

### Health equity

Health equity in metabolic and bariatric surgery is a pressing concern, as evidenced by findings from various studies revealing pervasive disparities in access, outcomes, and postoperative experiences. White patients with greater median incomes and private insurance are more likely to undergo metabolic and bariatric surgery and, therefore, are overrepresented in the medical literature [24, 25].

Socioeconomic factors are significant determinants influencing the accessibility of metabolic and bariatric surgery, overshadowing medical eligibility considerations [26–30]. The persistent disparities based on race, income, education level, and insurance type underscore the imperative for targeted public health efforts aimed at equalizing and expanding access to bariatric interventions.

Socioeconomic status not only influenced the likelihood of undergoing metabolic and bariatric surgery but also impacted postoperative outcomes. Studies have revealed higher risks of postoperative complications and readmissions for African Americans, as well as a higher rate of re-interventions for Indigenous persons compared to white patients [31, 32]. One study found differences in outcomes with regards to metabolic outcomes as well, with African American and Asian patients needing additional interventions to achieve similar glucose control as white patients [33].

While the guidelines provide valuable insights into the clinical aspects of metabolic and bariatric surgery, it is essential to consider the broader context of health disparities and diversity when implementing these recommendations. Further research and work is needed to address health equity and disparities related to metabolic and bariatric surgery and to develop a more comprehensive understanding of the condition's impact on diverse patient populations and to guide efforts toward more equitable healthcare practices.

Our patient representative also noted the frequent exclusion of patients with obesity from clinical trials; this is an important disparity across all areas of medicine as patients with obesity may be under- or over-dosed on the standard medication regimens. Their inclusion in clinical trials is essential to identify this issue and is of particular importance to KQ3 regarding IBD.

## Supplementary Information

Below is the link to the electronic supplementary material.

Supplementary file1 (DOCX 14 KB)

Supplementary file2 (DOCX 13 KB)

Supplementary file3 (DOCX 29 KB)

Supplementary file4 (DOCX 111 KB)

Supplementary file5 (PDF 190 KB)

Supplementary file6 (DOCX 20 KB)

Supplementary file7 (DOCX 37 KB)

## Abbreviations

BMI: Body mass index
CBD: Common bile duct
CI: Confidence interval
DS: Duodenal switch
EDGE: Endoscopic ultrasound directed transGastric ERCP
ERCP: Endoscopic retrograde cholangiopancreatography
GERD: Gastroesophageal reflux disease
GRADE: Grading of recommendations, assessment, development, and evaluations
IBD: Inflammatory bowel disease
IOC: Intraoperative cholangiography
KQ: Key question
MSA: Magnetic sphincter augmentation
PICO: Population, intervention, comparison, outcome
RCT: Randomized controlled trial
RYGB: Roux en Y Gastric Bypass
SAGES: The Society of American Gastrointestinal and Endoscopic Surgeons
