# Correction: SAGES guidelines for the management of comorbidities relevant to metabolic and bariatric surgery

**DOI:** 10.1007/s00464-025-11537-3

**Published:** 2025-01-13

**Authors:** Sunjay S. Kumar, Claire Wunker, Amelia Collings, Varun Bansal, Theofano Zoumpou, Julietta Chang, Noe Rodriguez, Andrew Sabour, Lisa Renee Hilton, Omar M. Ghanem, Bradley S. Kushner, Lindsey Jean Loss, Essa M. Aleassa, Ivy N. Haskins, Subhashini Ayloo, Adam Reid, David Wayne Overby, Peter Hallowell, Tammy Lyn Kindel, Bethany J. Slater, Francesco Palazzo

**Affiliations:** 1 Department of Surgery, Thomas Jefferson University Hospital, Philadelphia, PA USA; 2 Department of Surgery, Saint Louis University, St. Louis, MO USA; 3 Department of Surgery, University of Louisville, Louisville, KY USA; 4 Department of Surgery, University of Colorado, Aurora, CO USA; 5 Department of Surgery Rutgers, New Jersey Medical School, Newark, NJ USA; 6 Department of Surgery, Kaiser Permanente Bellevue Medical Center, Baldwin Park, CA USA; 7 Department of Surgery, Cleveland Clinic, Cleveland, OH USA; 8 Department of Surgery, University of Nevada, Las Vegas, NV USA; 9 Department of Surgery, Medical College of Georgia, Augusta, Georgia; 10 Department of Surgery, Mayo Clinic, Rochester, MN USA; 11 Department of Surgery, Washington University in St. Louis, St. Louis, MO USA; 12 Department of Surgery, Oregon Health and Science University, Portland, OR USA; 13 Department of Surgery, University of Nebraska Medical Center, Omaha, NE USA; 14 Department of Surgery, Brown University, Providence, RI USA; 15 Department of Surgery, Southern Illinois University School of Medicine, Springfield, IL USA; 16 Department of Surgery, University of North Carolina, Chapel Hill, NC USA; 17 Department of Surgery, University of Virginia, Charlottesville, VA USA; 18 Department of Surgery, Medical College of Wisconsin, Madison, WI USA; 19 Department of Surgery, University of Chicago, Chicago, IL USA

**Correction: Surgical Endoscopy (2024) 39:1–10** **https://doi.org/10.1007/s00464-024-11433-2**

The original online version of this article was revised to correct the presentation of the coauthor Theofano Zoumpou.

The original article has been corrected.

