# Supplementary material for: SAGES guidelines for the management of comorbidities relevant to metabolic and bariatric surgery
Source: Surg Endosc. 2024 Dec 11;39(1):1–10. doi: 10.1007/s00464-024-11433-2 (PMC11666733; doi:10.1007/s00464-024-11433-2)
Supplement: Supplementary file 1 — Supplementary file1 (DOCX 14 KB) [file 464_2024_11433_MOESM1_ESM.docx]

Sunjay S. Kumar, MD - data collection, non-voting panel member, manuscript drafting and revision

Claire Wunker, MD - data collection, manuscript revision

Amelia Collings, MD - data collection, manuscript revision

Varun Bansal, MBBS - data collection, manuscript revision

Theofano Zampou, MD - data collection, manuscript revision

Julietta Chang, MD FACS DABOM - Study design, data collection, panel member, manuscript revision

Noe Rodriguez, MD - Study design, data collection, panel member, manuscript revision

Andrew Sabour, MD - Study design, data collection, panel member, manuscript revision

Renee Hilton, MD - Study design, data collection, panel member, manuscript revision

Omar Ghanem, MD - Study design, data collection, panel member, manuscript revision

Bradley Kushner, MD - Study design, data collection, panel member, manuscript revision

Lindsey Jean Loss, MD - Study design, data collection, panel member, manuscript revision

Essa M. Aleassa, MD - Study design, data collection, panel member, manuscript revision

Ivy N. Haskins, MD - Study design, data collection, panel member, manuscript revision

Subhashini Ayloo, MD - Study design, data collection, panel member, manuscript revision

Adam Reid, MD - Study design, data collection, panel member, manuscript drafting and revision

David Wayne Overby, MD - Study design, data collection, panel member, manuscript revision

Peter Hallowell, MD - Study design, data collection, panel member, manuscript revision

Tammy Lyn Kindel, MD PhD - Study design, data collection, panel member, manuscript drafting and revision
Bethany J. Slater, MD - Study design, manuscript drafting and revision

Francesco Palazzo, MD – Study design, data collection, panel member, manuscript drafting and revision
