# Supplementary material for: SAGES guidelines for the management of comorbidities relevant to metabolic and bariatric surgery
Source: Surg Endosc. 2024 Dec 11;39(1):1–10. doi: 10.1007/s00464-024-11433-2 (PMC11666733; doi:10.1007/s00464-024-11433-2)
Supplement: Supplementary file 2 — Supplementary file2 (DOCX 13 KB) [file 464_2024_11433_MOESM2_ESM.docx]

DISCLOSURES:

Drs. Aleassa, Ayloo, Bansal, Chang, Collings, Ghanem, Hilton, Kumar, Kushner, Loss, Overby, Palazzo, Reid, Rodriguez, Sabour, Wunker, and Zoumpou report no disclosures.

Drs. Hallowell, Haskins, Kindel, and Slater report the following disclosures:

Dr. Hallowell - leadership roles within Piedmont Liability Trust (Board of Directors) and Midwest Surgical Association (President-elect)

Dr. Haskins - royalties from UpToDate

Dr. Kindel - grants from the National Heart, Lung, and Blood Institute and the American College of Surgeons, consulting fees from Johnson & Johnson, payment or honoraria from the Translational Medicine Academy, travel support from Medtronic and Intuitive, U.S. Provisional Patent Application No. 63/505,036, and an unpaid leadership role with the Treo Foundation

Dr. Slater - consulting fees from Hologic, leadership role in the SAGES Guidelines committee (Chair)
