# Supplementary material for: SAGES guidelines for the management of comorbidities relevant to metabolic and bariatric surgery
Source: Surg Endosc. 2024 Dec 11;39(1):1–10. doi: 10.1007/s00464-024-11433-2 (PMC11666733; doi:10.1007/s00464-024-11433-2)
Supplement: Supplementary file 3 — Supplementary file3 (DOCX 29 KB) [file 464_2024_11433_MOESM3_ESM.docx]

# Guidelines for the Management of Bariatric Surgeries

# Literature Searches Summary Chart

(Updated: 7/27/23)

| **Summary - Run Dates:** 7/22/2020 and 2/26/2022  **Librarian:** Holly Ann Burt |
| --- |

| **OVERALL**  **SEARCH**  **RESULTS** | Total records identified by database searches | 4,073 |
| --- | --- | --- |
|  | Records located by handsearching trials, databases, and references | 71 |
|  | Total trials identified in searches | 353 |
|  | Total duplicates identified (across KQs, by HAB, in Endnote, in Covidence) | 2,171 |
|  | Total unique records screened | 1,620 |

| **Database searched (Coverage)** | | |
| --- | --- | --- |
| **Systematic Reviews**  Cochrane Library/ Cochrane (2009-2023) | **Clinical Trials**  Clinicaltrials.gov/NLM (2000-2023) | **Medicine**  Embase/Elsevier (1947-2023)  [PubMed](https://www.ncbi.nlm.nih.gov/pubmed/10653237)/NLM (1809/1966-2023) |
| Databases handsearched for articles published by the trials retrieved by Clinicaltrials.gov and Cochrane searches: ICTRP (International Clinical Trials Registry Platform)/WHO, Clinicaltrials.gov, PubMed, and Google Scholar | | |

## KQ1

| 1. **Should routine IOC versus alternative options be used for patients undergoing cholecystectomy with current or previous gastrointestinal bypass type bariatric procedures (RYGB and DS, etc.)?** | | |
| --- | --- | --- |
| **Database** | **Final search strategies** | **Results** |
| PubMed  Q1 | ((cholecystectomy [mh] OR cholecystectomy [tw] OR cholecystectomies [tw]) AND (Bariatric Surgery [mh] OR "bariatric surgery" [tw] OR gastric bypass[tw] OR Gastroenterostomy[mh] OR Roux-en-Y[tw] OR "Anastomosis, Roux-en-Y"[mh] OR OAGB[tw] OR "anastomosis gastric"[tw] OR duodenum/surgery[mh] OR duodenal switch[tw] OR SADI[tw] OR "single anastomosis"[tw] OR Gastroenterostomy[mh]) AND (Clinical Study[pt] OR Comparative Study[pt] OR Epidemiologic Studies[mh] OR Evaluation Study[pt] OR Meta-Analysis[pt] OR Multicenter Study[pt] OR Systematic Review[pt] OR randomized[tiab] OR study[tiab] OR studies [tiab] OR follow-up studies[mh] OR Practice Guideline[pt] OR "practice guidelines as topic"[mh] OR guideline[title] OR guidelines[title])) NOT (Case reports[pt] OR "case report"[tw] OR ("animals"[MH:noexp] NOT "humans"[MH]) OR rat[tiab] OR rats[tiab] OR dog[tiab] OR dogs[tiab] OR porcine[tiab] OR "infant"[MeSH] OR (child[mh] NOT adult[mh]) OR children[title] OR child[title] OR infants[title] OR infant[title] OR pediatric [tiab] OR (1889:1989 [ppdat])) | 537 |
| Embase  Q1 | (('cholecystectomy'/exp OR cholecystectomy) AND ('bariatric surgery'/exp OR 'bariatric surgery' OR 'roux en y' OR 'roux y anastomosis'/exp OR 'roux y anastomosis' OR 'roux-en-y gastric bypass'/exp OR 'roux-en-y gastric bypass' OR 'biliopancreatic bypass'/exp OR 'biliopancreatic bypass' OR 'duodenal switch' OR 'single anastomosis gastric bypass'/exp OR 'single anastomosis gastric bypass') AND ([adult]/lim OR [aged]/lim OR [middle aged]/lim OR [very elderly]/lim OR [young adult]/lim) AND ('practice guideline'/exp OR 'practice guideline' OR [cochrane review]/lim OR [systematic review]/lim OR [meta analysis]/lim OR [clinical study]/lim)) NOT ([conference abstract]/lim OR 'case report'/exp OR 'case report' OR [animals]/lim OR [1889-1989]/py) | 586 |
| Cochrane  Q1 | cholecystectomy AND (bariatric surgery OR Gastric bypass OR Roux-en-y OR SADI OR OAGB) | 66 |
| Clinical Trials Q1 | cholecystectomy AND (bariatric surgery OR gastric bypass OR SADI OR OAGB OR duodenum OR roux-en-y) | 42 |
| PubMed Run 2 | Above NOT ("1960/01/01"[CRDT] : "2020/07/20"[CRDT]) | 56 |
| Embase Run 2 | Above NOT [1889-2019]/py | 169 |
| Cochrane Run 2 | Above with Cochrane Library publication date from Jul 2020 to Feb 2022 | 16 |
| Clinical Trials Run 2 | cholecystectomy AND (bariatric surgery OR gastric bypass OR SADI OR OAGB OR duodenum OR roux-en-y) \| Active, not recruiting, Completed, Suspended, Terminated, Withdrawn, Unknown status | 40 |
| **Q1**  **SEARCH RESULTS** | Total records identified by database searches | 1,512 |
|  | Additional records located by handsearching | 13 |
|  | Total trials identified in searches | 115 |
|  | Total duplicates found | 401 |
|  | **Total records screened** | **1,009** |

| 1. **In patients with GERD status post Laparoscopic Sleeve Gastrectomy (LSG) should surgical or medical therapy be used?** | | | |
| --- | --- | --- | --- |
| **Database** | **Final search strategies** | | **Results** |
| PubMed Q4 | (("gastroesophageal reflux"[mh] OR gerd[tw] OR Gastroesophageal Reflux[tw]) AND ("Sleeve gastrectomy"[tiab] OR "Sleeve gastrectomy" [ot] OR (Gastrectomy[mh] AND Sleeve [tw])) AND (Clinical Study[pt] OR Comparative Study[pt] OR "Epidemiologic Studies" [mh] OR Evaluation Study[pt] OR Meta-Analysis[pt] OR Multicenter Study[pt] OR Systematic Review[pt] OR randomized[tiab] OR study[tiab] OR studies [tiab] OR follow-up studies[mh] OR Practice Guideline[pt] OR "practice guidelines as topic"[mh] OR guideline[title] OR guidelines[title])) NOT ("Case reports"[pt] OR "case report"[tw] OR ("animals"[MH:noexp] NOT "humans"[MH]) OR rat[tiab] OR rats[tiab] OR dog[tiab] OR dogs[tiab] OR porcine[tiab] OR "infant"[MeSH] OR (child[mh] NOT adult[mh]) OR children[title] OR child[title] OR infants[title] OR infant[title] OR pediatric [tiab] OR "1965:1989" [ppdat]) | | 356 |
| Embase  Q4 | (('gastroesophageal reflux'/exp OR 'gastroesophageal reflux') AND ('sleeve gastrectomy'/exp OR 'sleeve gastrectomy') AND ([adult]/lim OR [aged]/lim OR [middle aged]/lim OR [very elderly]/lim OR [young adult]/lim) AND ('practice guideline'/exp OR 'practice guideline' OR [cochrane review]/lim OR [systematic review]/lim OR [meta analysis]/lim OR [clinical study]/lim)) NOT ([conference abstract]/lim OR 'case report'/exp OR 'case report' OR [animals]/lim OR [1889-1989]/py) | | 450 |
| Cochrane Q4 | (Sleeve gastrectomy) AND (GERD OR "gastroesophageal Reflux") | | 76 |
| Clinical Trials Q4 | Sleeve Gastrectomy \| Gastroesophageal Reflux OR GERD | | 26 |
| PubMed | Above NOT ("1960/01/01"[CRDT] : "2020/07/20"[CRDT]) | | 133 |
| Embase | Above NOT [1889-2019]/py | | 263 |
| Cochrane | Above with Cochrane Library publication date from Jun 2020 to Feb 2022 | | 24 |
| Clinical Trials | Sleeve Gastrectomy \| Active, not recruiting, Completed, Suspended, Terminated, Withdrawn, Unknown status Studies \| Gastroesophageal Reflux OR GERD | | 16 |
| **Q2**  **SEARCH RESULTS** | Total records identified by database searches | | 1,340 |
|  | Additional records located by handsearching | | 20 |
|  | Total trials identified in searches | | 76 |
|  | Total duplicates found | | 484 |
|  | **Total records screened** | | **800** |
| 1. **Should SG or intestinal bypass procedures be used in obese patients with inflammatory bowel disease (IBD)?** | | | |
| **Database** | **Final search strategies** | **Results** | |
| PubMed Q3 | (("Crohn’s disease" [tw] OR "Crohn disease" [tw] OR "Inflammatory Bowel Diseases"[mh] OR IBD[tiab] OR IBD [ot] OR "Ulcerative Colitis"[tw]) AND ("Bariatric Surgery "[mh] OR "bariatric surgery" [tw] OR "gastric bypass"[tw] OR Gastroenterostomy[mh] OR "Roux-en-Y"[tw] OR "Anastomosis, Roux-en-Y"[mh] OR OAGB[tw] OR "anastomosis gastric"[tw] OR duodenum/surgery[mh] OR "duodenal switch"[tw] OR SADI[tw] OR "single anastomosis"[tw] OR Gastroenterostomy[mh] OR "Sleeve gastrectomy"[tiab] OR "Sleeve gastrectomy" [ot] OR (Gastrectomy[mh] AND Sleeve [tw])) AND (Clinical Study[pt] OR Comparative Study[pt] OR "Epidemiologic Studies" [mh] OR Evaluation Study[pt] OR Meta-Analysis[pt] OR Multicenter Study[pt] OR Systematic Review[pt] OR randomized[tiab] OR study[tiab] OR studies [tiab] OR follow-up studies[mh] OR Practice Guideline[pt] OR "practice guidelines as topic"[mh] OR guideline[title] OR guidelines[title]) ) NOT ("Case reports"[pt] OR "case report"[tw] OR ("animals"[MH:noexp] NOT "humans"[MH]) OR rat[tiab] OR rats[tiab] OR dog[tiab] OR dogs[tiab] OR porcine[tiab] OR "infant"[MeSH] OR (child[mh] NOT adult[mh]) OR children[title] OR child[title] OR infants[title] OR infant[title] OR pediatric [tiab] OR "1965:1989" [ppdat]) | 64 | |
| Embase  Q3 | ('ulcerative colitis'/exp OR 'ulcerative colitis' OR 'inflammatory bowel disease'/exp OR 'inflammatory bowel disease' OR 'crohn disease'/exp OR 'crohn disease') AND ('bariatric surgery'/exp OR 'bariatric surgery' OR 'roux en y' OR 'roux y anastomosis'/exp OR 'roux y anastomosis' OR 'roux-en-y gastric bypass'/exp OR 'roux-en-y gastric bypass' OR 'biliopancreatic bypass'/exp OR 'biliopancreatic bypass' OR 'duodenal switch' OR 'single anastomosis gastric bypass'/exp OR 'single anastomosis gastric bypass') AND ([adult]/lim OR [aged]/lim OR [middle aged]/lim OR [very elderly]/lim OR [young adult]/lim) AND ('practice guideline'/exp OR 'practice guideline' OR [cochrane review]/lim OR [systematic review]/lim OR [meta analysis]/lim OR [clinical study]/lim)) NOT ([conference abstract]/lim OR 'case report'/exp OR 'case report' OR [animals]/lim OR [1889-1989]/py) | 52 | |
| Cochrane Q3 | (Crohn disease OR Ulcerative Colitis OR "Inflammatory Bowel Disease" OR IBD) AND ("bariatric surgery" OR "Gastric bypass" OR Roux-en-y OR SADI OR OAGB OR "Sleeve Gastrectomy") | 10 | |
| Clinical Trials Q3 | (bariatric surgery OR gastric bypass OR SADI OR OAGB OR duodenum OR roux-en-y OR Sleeve Gastrectomy) \| Ulcerative Colitis OR Crohn Disease OR IBD | 46 | |
| PubMed | Above NOT ("1960/01/01"[CRDT] : "2020/07/20"[CRDT]) | 10 | |
| Embase | Above NOT [1889-2019]/py | 31 | |
| Cochrane | Above; with Cochrane Library publication date from Jun 2020 to Feb 2022 | 3 | |
| Clinical Trials | (bariatric surgery OR gastric bypass OR SADI OR OAGB OR duodenum OR roux-en-y OR Sleeve Gastrectomy) \| Active, not recruiting, Completed, Suspended, Terminated, Withdrawn, Unknown status Studies \| Ulcerative Colitis OR Crohn Disease OR IBD | 38 | |
| **Q3**  **SEARCH RESULTS** | Total records identified by database searches | 254 | |
|  | Additional records located by handsearching | 1 | |
|  | Total trials identified in searches | 85 | |
|  | Total duplicates found | 54 | |
|  | **Total records screened** | **116** | |
