# Supplementary material for: SAGES guidelines for the management of comorbidities relevant to metabolic and bariatric surgery
Source: Surg Endosc. 2024 Dec 11;39(1):1–10. doi: 10.1007/s00464-024-11433-2 (PMC11666733; doi:10.1007/s00464-024-11433-2)
Supplement: Supplementary file 4 — Supplementary file4 (DOCX 111 KB) [file 464_2024_11433_MOESM4_ESM.docx]

**Guidelines for Bariatric Surgery**

**PRISMA Flow Diagram**

**KQ1**

Records identified through database searching

ClinicalTrials.gov/NLM (n = 275)

Cochrane Library/Cochrane (n = 238)

Embase/Elsevier (n = 1,991)

PubMed/NLM (n = 1,569)

(n = 4,073) including KQ1 (n = 1,512)

Additional records identified through handsearching trials and citation searching

ClinicalTrials.gov/NLM (n = 24)

PubMed (hand identified) (n = 22)

Citation searching (n = 25)

(n = 71) including KQ1 (n= 13)

## Literature Search

Records removed

Duplicates identified during handsearching (n = 5)

Duplicates identified by Endnotes (n = 1,854)

Duplicates identified manually in Endnotes (n = 144)

Duplicates identified by Covidence (n = 164)

Duplicates identified manually in Covidence (n = 4)

Trials (n = 353)

(n = 2,171) including KQ1 (n = 516)

Records not identified as KQ1
(n = 611)

Unique records

(n = 1,620) including KQ1 (n = 1,512)

## Screening

KQ1 records screened
(n = 1,009)

Records excluded
(n = 957)

Full-text articles excluded

(n = 43)

23 Wrong intervention

9 Wrong patient population

5 Wrong outcomes

2 Wrong study design

2 SR - Citations searched

1 Conference abstract

1 Non-English

Full-text articles assessed for eligibility
(n = 52)

52

## Eligibility

## Included

Studies included in appraisal/ guideline/recommendations
(n = 9)

See Literature Searches Summary document for search details.

**Guidelines for Bariatric Surgery**

**PRISMA Flow Diagram**

**KQ2**

Records identified through database searching

ClinicalTrials.gov/NLM (n = 275)

Cochrane Library/Cochrane (n = 238)

Embase/Elsevier (n = 1,991)

PubMed/NLM (n = 1,569)

(n = 4,073) including KQ4 (n = 1,340)

Additional records identified through handsearching trials and citation searching

ClinicalTrials.gov/NLM (n = 24)

PubMed (hand identified) (n = 22)

Citation searching (n = 25)

(n = 71) including KQ4 (n= 20)

## Literature Search

Records removed

Duplicates identified during handsearching (n = 5)

Duplicates identified by Endnotes (n = 1,854)

Duplicates identified manually in Endnotes (n = 144)

Duplicates identified by Covidence (n = 164)

Duplicates identified manually in Covidence (n = 4)

Trials (n = 353)

(n = 2,171) including KQ4 (n = 560)

Records not identified as KQ4
(n = 819)

Unique records

(n = 1,620) including KQ4 (n = 800)

Records excluded
(n = 670)

## Screening

KQ4 records screened
(n = 801)

Full-text articles excluded

(n = 91)

32 Wrong outcomes

25 Wrong patient population

11 SR - Citations searched

10 Wrong study design

6 Wrong comparator

4 Wrong intervention

2 Non-English

1 review article no data

Full-text articles assessed for eligibility
(n = 130)

21)

52

## Eligibility

## Included

Studies included in appraisal/ guideline/recommendations
(n = 39)

See Literature Searches Summary document for search details.

**Guidelines for Bariatric Surgery**

**PRISMA Flow Diagram**

**KQ3**

Records identified through database searching

ClinicalTrials.gov/NLM (n = 275)

Cochrane Library/Cochrane (n = 238)

Embase/Elsevier (n = 1,991)

PubMed/NLM (n = 1,569)

(n = 4,073) including KQ3 (n = 254)

Additional records identified through handsearching trials and citation searching

ClinicalTrials.gov/NLM (n = 24)

PubMed (hand identified) (n = 22)

Citation searching (n = 25)

(n = 71) including KQ3 (n= 1)

## Literature Search

Records removed

Duplicates identified during handsearching (n = 5)

Duplicates identified by Endnotes (n = 1,854)

Duplicates identified manually in Endnotes (n = 144)

Duplicates identified by Covidence (n = 164)

Duplicates identified manually in Covidence (n = 4)

Trials (n = 353)

(n = 2,171) including KQ3 (n = 139)

Records not identified as KQ3
(n = 1,504)

Unique records

(n = 1,620) including KQ3 (n = 116)

Records excluded
(n = 85)

## Screening

KQ3 records screened
(n = 116)

Full-text articles excluded

(n = 20)

12 SR - Citations searched

7 Wrong patient population

1 Wrong intervention

Full-text articles assessed for eligibility
(n = 31)

52

## Eligibility

## Included

Studies included in appraisal/ guideline/recommendations
(n = 11)

See Literature Searches Summary document for search details.
