# Supplementary material for: SAGES guidelines for the management of comorbidities relevant to metabolic and bariatric surgery
Source: Surg Endosc. 2024 Dec 11;39(1):1–10. doi: 10.1007/s00464-024-11433-2 (PMC11666733; doi:10.1007/s00464-024-11433-2)
Supplement: Supplementary file 5 — Supplementary file5 (PDF 190 KB) [file 464_2024_11433_MOESM5_ESM.pdf]

Figures

Figure 1 (Analysis 1.3)

	Sleeve		Bypass			Odds Ratio
**Study or Subgroup**	**Events**	**Total**	**Events**	**Total**	**Weight**	**M-H, Random, 95% CI**
Hudson 2019	0	9	1	3	10.0%	0.09 [0.00, 2.88]
Aelfers 2018	1	23	2	9	18.9%	0.16 [0.01, 2.03]
Reenaers 2021	7	73	1	3	19.2%	0.21 [0.02, 2.65]
Heshmati 2019	1	35	2	19	20.0%	0.25 [0.02, 2.96]
Aminian 2016	3	9	4	8	31.8%	0.50 [0.07, 3.55]
**Total (95% CI)**		**149**		**42**	**100.0%**	**0.25 [0.08, 0.75]**
Total events	12		10			

Heterogeneity: Tau² = 0.00; Chi² = 0.96, df = 4 (P = 0.92); I² = 0%
Test for overall effect: Z = 2.46 (P = 0.01)

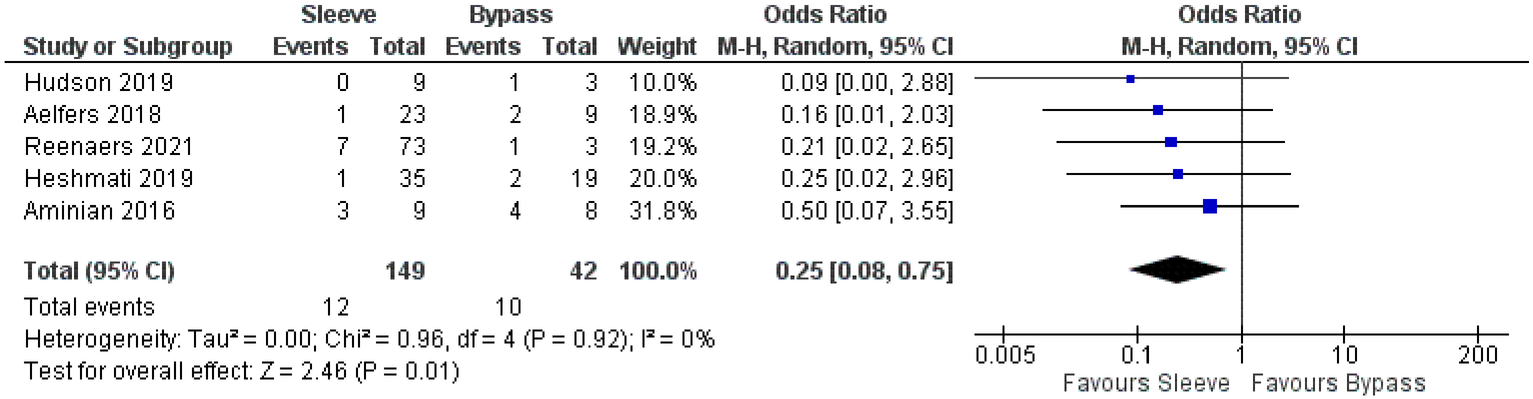

Forest plot of comparison: 1 Sleeve vs Bypass, outcome: 1.3 Perioperative complications (<30d) Clavien dindo ≥ 2 – yes/no.

Figure 2 (Analysis 1.4)

	Sleeve		Bypass			Odds Ratio
**Study or Subgroup**	**Events**	**Total**	**Events**	**Total**	**Weight**	**M-H, Random, 95% CI**
Hudson 2019	0	9	1	3	14.7%	0.09 [0.00, 2.88]
Aminian 2016	0	9	2	8	17.6%	0.14 [0.01, 3.34]
Reenaers 2021	2	73	0	3	17.3%	0.24 [0.01, 6.13]
Heshmati 2019	2	35	3	19	50.4%	0.32 [0.05, 2.13]
**Total (95% CI)**		**126**		**33**	**100.0%**	**0.22 [0.06, 0.83]**
Total events	4		6			

Heterogeneity: Tau² = 0.00; Chi² = 0.52, df = 3 (P = 0.92); I² = 0%
Test for overall effect: Z = 2.22 (P = 0.03)

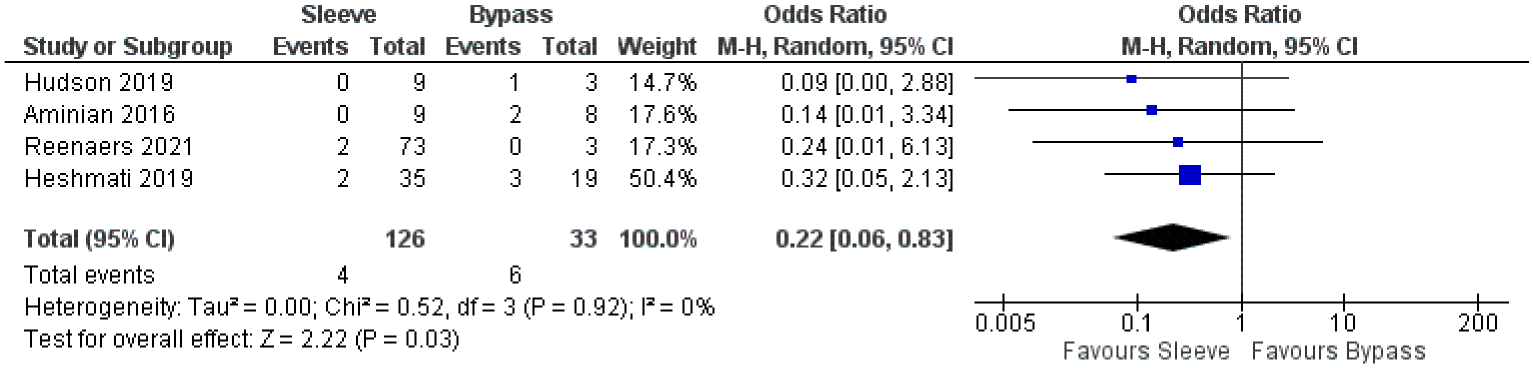

Forest plot of comparison: 1 Sleeve vs Bypass, outcome: 1.4 Long term complications (dumping syndrome, malabsorption, leaks, fistulas, etc) – yes/no.

Figure 3 (Analysis 1.5)

Study or Subgroup	Sleeve Events	Sleeve Total	Bypass Events	Bypass Total	Weight	Odds Ratio M-H, Random, 95% CI
Aminian 2016	0	9	0	8		Not estimable
Hudson 2019	0	9	0	3		Not estimable
Heshmati 2019	1	35	4	19	100.0%	0.11 [0.01, 1.07]
**Total (95% CI)**		**53**		**30**	**100.0%**	**0.11 [0.01, 1.07]**
Total events	1		4			
Heterogeneity: Not applicable						
Test for overall effect: Z = 1.90 (P = 0.06)						

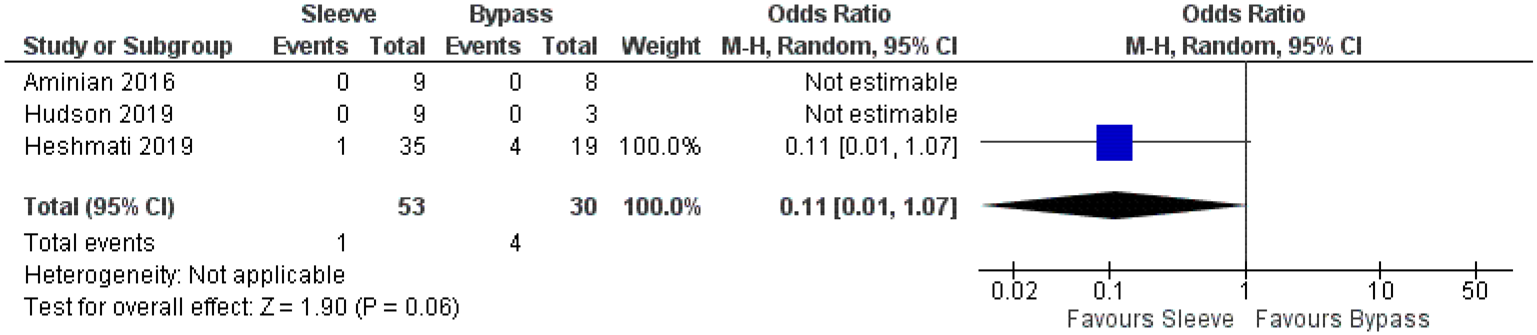

Forest plot of comparison: 1 Sleeve vs Bypass, outcome: 1.5 IBD Worsening (Pain requiring medical therapy) – yes/no.

Figure 4 (Analysis 1.6)

Study or Subgroup	Sleeve Events	Sleeve Total	Bypass Events	Bypass Total	Weight	Odds Ratio M-H, Random, 95% CI
McKenna 2020	0	14	0	14		Not estimable
Aminian 2016	0	9	0	8		Not estimable
Heshmati 2019	2	35	3	19	100.0%	0.32 [0.05, 2.13]
**Total (95% CI)**		**58**		**41**	**100.0%**	**0.32 [0.05, 2.13]**
Total events	2		3			
Heterogeneity: Not applicable						
Test for overall effect: Z = 1.17 (P = 0.24)						

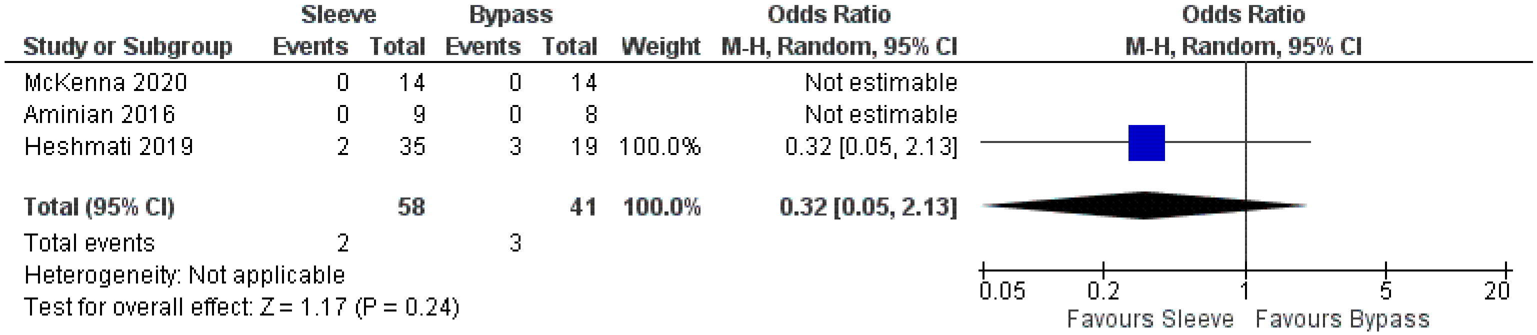

Forest plot of comparison: 1 Sleeve vs Bypass, outcome: 1.6 IBD Worsening (Obstruction, hemorrhage, fistula, or perforation, combined if reported separately) – yes/no.

Figure 5 (Analysis 1.7)

Study or Subgroup	Sleeve Events	Sleeve Total	Bypass Events	Bypass Total	Weight	Odds Ratio M-H, Random, 95% CI
Aminian 2016	0	9	0	8		Not estimable
Aelfers 2018	0	23	1	9	100.0%	0.12 [0.00, 3.25]
**Total (95% CI)**		**32**		**17**	**100.0%**	**0.12 [0.00, 3.25]**
Total events	0		1			

Heterogeneity: Not applicable
Test for overall effect: Z = 1.26 (P = 0.21)

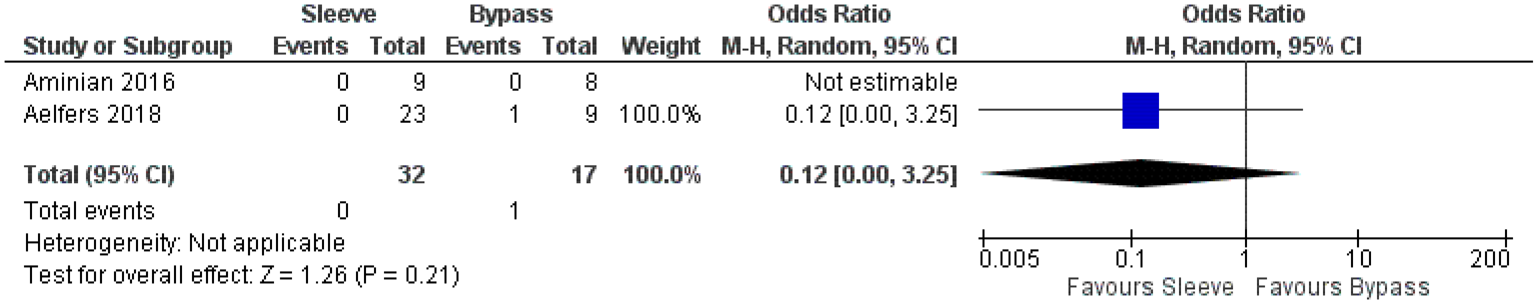

Forest plot of comparison: 1 Sleeve vs Bypass, outcome: 1.7 IBD Worsening (Ulceration) − yes/no.

Figure 6 (Analysis 1.8)

Study or Subgroup	Sleeve Events	Sleeve Total	Bypass Events	Bypass Total	Weight	Odds Ratio M-H, Random, 95% CI
Heshmati 2019	1	35	7	19	54.5%	0.05 [0.01, 0.45]
Aelfers 2018	2	23	0	9	45.5%	2.21 [0.10, 50.58]
**Total (95% CI)**		**58**		**28**	**100.0%**	**0.28 [0.01, 11.33]**
Total events	3		7			

Heterogeneity: $Tau^2 = 5.27$; $Chi^2 = 3.77$, df = 1 (P = 0.05); $I^2 = 73\%$
Test for overall effect: Z = 0.67 (P = 0.50)

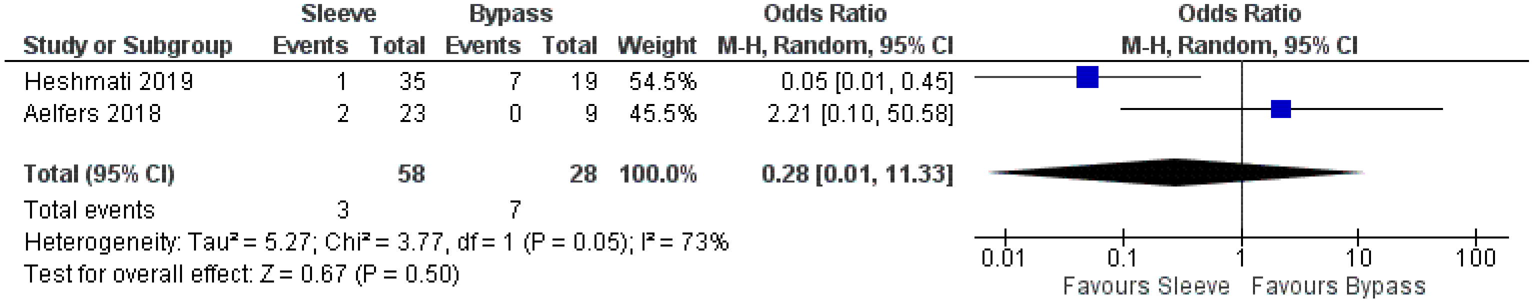

Forest plot of comparison: 1 Sleeve vs Bypass, outcome: 1.8 IBD Worsening (patient reported) − yes/no.

Figure 7 (Analysis 1.10)

Study or Subgroup	Sleeve Events	Sleeve Total	Bypass Events	Bypass Total	Weight	Odds Ratio M-H, Random, 95% CI
Aelfers 2018	0	23	0	9		Not estimable
Reenaers 2021	0	73	0	3		Not estimable
McKenna 2020	0	14	0	14		Not estimable
Hudson 2019	0	9	0	3		Not estimable
Heshmati 2019	1	35	0	19	51.4%	1.70 [0.07, 43.66]
Aminian 2016	1	9	0	8	48.6%	3.00 [0.11, 84.56]
**Total (95% CI)**		**163**		**56**	**100.0%**	**2.24 [0.22, 22.96]**
Total events	2		0			

Heterogeneity: Tau² = 0.00; Chi² = 0.06, df = 1 (P = 0.81); I² = 0%
Test for overall effect: Z = 0.68 (P = 0.50)

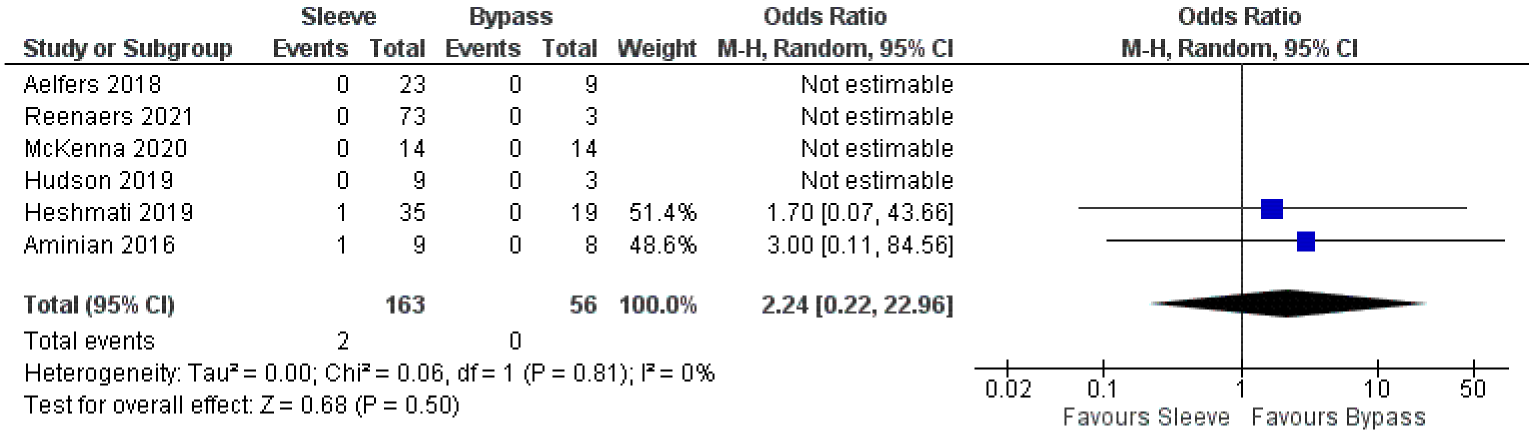

Forest plot of comparison: 1 Sleeve vs Bypass, outcome: 1.10 Mortality (all cause) – yes/no.

Figure 8 (Analysis 1.11)

Study or Subgroup	Sleeve Events	Sleeve Total	Bypass Events	Bypass Total	Weight	Odds Ratio M-H, Random, 95% CI
Hudson 2019	0	9	0	3		Not estimable
Aminian 2016	0	9	0	9		Not estimable
Aelfers 2018	0	23	2	9	17.0%	0.06 [0.00, 1.48]
Reenaers 2021	4	73	1	3	23.6%	0.12 [0.01, 1.57]
McKenna 2020	0	14	2	14	17.1%	0.17 [0.01, 3.94]
Heshmati 2019	4	35	2	19	42.3%	1.10 [0.18, 6.62]
**Total (95% CI)**		**163**		**57**	**100.0%**	**0.29 [0.07, 1.15]**
Total events	8		7			

Heterogeneity: Tau² = 0.32; Chi² = 3.55, df = 3 (P = 0.31); I² = 16%
Test for overall effect: Z = 1.76 (P = 0.08)

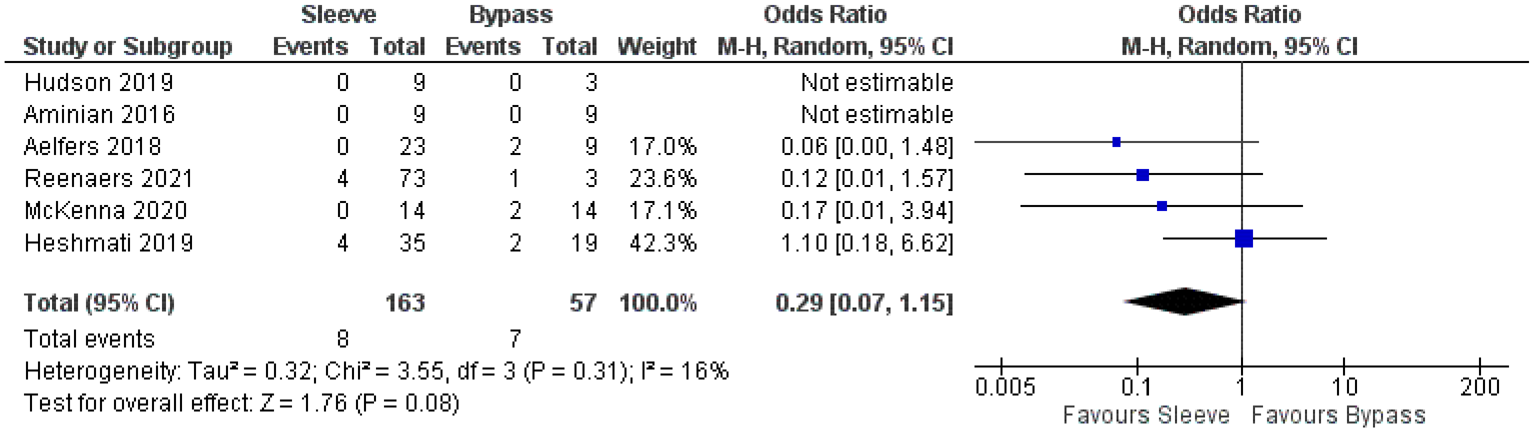

Forest plot of comparison: 1 Sleeve vs Bypass, outcome: 1.11 Reoperations (failure of primary bariatric procedure, IBD revisional surgery, or both) – yes/no.
