# Supplementary material for: SAGES guidelines for the management of comorbidities relevant to metabolic and bariatric surgery
Source: Surg Endosc. 2024 Dec 11;39(1):1–10. doi: 10.1007/s00464-024-11433-2 (PMC11666733; doi:10.1007/s00464-024-11433-2)
Supplement: Supplementary file 6 — Supplementary file6 (DOCX 20 KB) [file 464_2024_11433_MOESM6_ESM.docx]

**Author(s):**

**Question:** Sleeve compared to Bypass in obese patients with inflammatory bowel disease (IBD)

**Setting:**

**Bibliography:** . [Intervention] for [health problem]. Cochrane Database of Systematic Reviews [Year], Issue [Issue].

| **Certainty assessment** | | | | | | | **№ of patients** | | **Effect** | | **Certainty** | **Importance** |
| --- | --- | --- | --- | --- | --- | --- | --- | --- | --- | --- | --- | --- |
| **№ of studies** | **Study design** | **Risk of bias** | **Inconsistency** | **Indirectness** | **Imprecision** | **Other considerations** | **Sleeve** | **Bypass** | **Relative (95% CI)** | **Absolute (95% CI)** |  |  |
| **Perioperative complications (<30d) Clavien dindo ≥2 – yes/no** | | | | | | | | | | | | |
| 5 | observational studies | serious^a^ | not serious | not serious | serious^b^ | strong association | 12/149 (8.1%) | 10/42 (23.8%) | **OR 0.25** (0.08 to 0.75) | **166 fewer per 1,000** (from 214 fewer to 48 fewer) | ⨁◯◯◯ Very low |  |
| **Long term complications (dumping syndrome, malabsorption, leaks, fistulas, etc) – yes/no** | | | | | | | | | | | | |
| 4 | observational studies | serious^a^ | not serious | not serious | serious^b^ | strong association | 4/126 (3.2%) | 6/33 (18.2%) | **OR 0.22** (0.06 to 0.83) | **135 fewer per 1,000** (from 169 fewer to 26 fewer) | ⨁◯◯◯ Very low |  |
| **IBD Worsening (Pain requiring medical therapy) – yes/no** | | | | | | | | | | | | |
| 3 | observational studies | serious^a^ | not serious | not serious | very serious^b,c^ | none | 1/53 (1.9%) | 4/30 (13.3%) | **OR 0.11** (0.01 to 1.07) | **117 fewer per 1,000** (from 132 fewer to 8 more) | ⨁◯◯◯ Very low |  |
| **IBD Worsening (Obstruction, hemorrhage, fistula, or perforation, combined if reported separately) – yes/no** | | | | | | | | | | | | |
| 3 | observational studies | serious^a^ | not serious | not serious | very serious^b,c^ | none | 2/58 (3.4%) | 3/41 (7.3%) | **OR 0.32** (0.05 to 2.13) | **49 fewer per 1,000** (from 69 fewer to 71 more) | ⨁◯◯◯ Very low |  |
| **IBD Worsening (Ulceration) – yes/no** | | | | | | | | | | | | |
| 2 | observational studies | serious^a^ | not serious | not serious | very serious^b,c^ | none | 0/32 (0.0%) | 1/17 (5.9%) | **OR 0.12** (0.00 to 3.25) | **51 fewer per 1,000** (from -- to 110 more) | ⨁◯◯◯ Very low |  |
| **IBD Worsening (patient reported) – yes/no** | | | | | | | | | | | | |
| 1 | observational studies | serious^d^ | not serious | not serious | very serious^b,c^ | none | 1/35 (2.9%) | 4/19 (21.1%) | **OR 0.11** (0.01 to 1.07) | **182 fewer per 1,000** (from 208 fewer to 11 more) | ⨁◯◯◯ Very low |  |
| **Mortality (all cause) – yes/no** | | | | | | | | | | | | |
| 6 | observational studies | serious^a^ | not serious | not serious | very serious^b,c^ | none | 2/163 (1.2%) | 0/56 (0.0%) | **OR 2.24** (0.22 to 22.96) | **0 fewer per 1,000** (from 0 fewer to 0 fewer) | ⨁◯◯◯ Very low |  |
|  |  |  |  |  |  |  |  | 5.7% |  | **62 more per 1,000** (from 44 fewer to 524 more) |  |  |
| **Reoperations (failure of primary bariatric procedure, IBD revisional surgery, or both) – yes/no** | | | | | | | | | | | | |
| 6 | observational studies | serious^a^ | not serious | not serious | very serious^b,c^ | none | 8/163 (4.9%) | 7/57 (12.3%) | **OR 0.29** (0.07 to 1.15) | **84 fewer per 1,000** (from 113 fewer to 16 more) | ⨁◯◯◯ Very low |  |

**CI:** confidence interval; **OR:** odds ratio

#### Explanations

a. The included studies were deemed unclear to high risk of bias due to concerns over patients selection, comparability of the two groups, and variable follow up periods.

b. This outcome had a small sample size. The estimate of the effect is fragile.

c. The relative effect estimate for this outcome crosses the threshold of significance.

d. The included study was deemed unclear risk of bias on the basis of patient selection, comparability, and follow up period.
