# Supplementary material for: SAGES guidelines for the management of comorbidities relevant to metabolic and bariatric surgery
Source: Surg Endosc. 2024 Dec 11;39(1):1–10. doi: 10.1007/s00464-024-11433-2 (PMC11666733; doi:10.1007/s00464-024-11433-2)
Supplement: Supplementary file 7 — Supplementary file7 (DOCX 37 KB) [file 464_2024_11433_MOESM7_ESM.docx]

| Question | |
| --- | --- |
| **Should Sleeve vs. Bypass be used for obese patients with inflammatory bowel disease (IBD)?** | |
| **Population:** | obese patients with inflammatory bowel disease (IBD) |
| **Intervention:** | Sleeve |
| **Comparison:** | Bypass |
| **Main outcomes:** | - Short-term postoperative complications (overall) - Long-term complications (dumping syndrome, malabsorption, leaks, fistula etc) - IBD improvement/worsening   - - Pain requiring medical therapy     - obstruction, hemorrhage, perforation     - Ulceration     - Stenosis     - overall QoL & GI specific QoL     - GI symptoms (diarrhea, constipation)     - Weight loss     - hospitalization or rescue treatment due to relapse - Drug related SAEs (Cushingoid, opportunistic infections, new malignancies) - Death - Reoperations (failure of primary bariatric procedure, IBD revisional surgery, or both |
| **Setting:** |  |
| **Perspective:** |  |
| **Background:** |  |
| **Conflict of interests:** |  |

# Assessment

| Problem Is the problem a priority? | | |
| --- | --- | --- |
| Judgement | Research evidence | Additional considerations |
| ○ No ○ Probably no ○ Probably yes ○ Yes ○ Varies ○ Don't know |  |  |
| Desirable Effects How substantial are the desirable anticipated effects? | | |
| Judgement | Research evidence | Additional considerations |
| ○ Trivial ○ Small ○ Moderate ○ Large ○ Varies ○ Don't know | \| **Outcomes** \| **№ of participants (studies) Follow-up** \| **Certainty of the evidence (GRADE)** \| **Relative effect (95% CI)** \| **Anticipated absolute effects^*^ (95% CI)** \| \| \| --- \| --- \| --- \| --- \| --- \| --- \| \| **Risk with Bypass** \| **Risk difference with Sleeve** \| \| Perioperative complications (<30d) Clavien dindo ≥2 – yes/no \| 191 (5 observational studies) \| ⨁◯◯◯ Very low^a,b^ \| **OR 0.25** (0.08 to 0.75) \| Study population \| \| \| 238 per 1,000 \| **166 fewer per 1,000** (214 fewer to 48 fewer) \| \| Long term complications (dumping syndrome, malabsorption, leaks, fistulas, etc) – yes/no \| 159 (4 observational studies) \| ⨁◯◯◯ Very low^a,b^ \| **OR 0.22** (0.06 to 0.83) \| Study population \| \| \| 182 per 1,000 \| **135 fewer per 1,000** (169 fewer to 26 fewer) \| \| IBD Worsening (Pain requiring medical therapy) – yes/no \| 83 (3 observational studies) \| ⨁◯◯◯ Very low^a,b,c^ \| **OR 0.11** (0.01 to 1.07) \| Study population \| \| \| 133 per 1,000 \| **117 fewer per 1,000** (132 fewer to 8 more) \| \| IBD Worsening (Obstruction, hemorrhage, fistula, or perforation, combined if reported separately) – yes/no \| 99 (3 observational studies) \| ⨁◯◯◯ Very low^a,b,c^ \| **OR 0.32** (0.05 to 2.13) \| Study population \| \| \| 73 per 1,000 \| **49 fewer per 1,000** (69 fewer to 71 more) \| \| IBD Worsening (Ulceration) – yes/no \| 49 (2 observational studies) \| ⨁◯◯◯ Very low^a,b,c^ \| **OR 0.12** (0.00 to 3.25) \| Study population \| \| \| 59 per 1,000 \| **51 fewer per 1,000** (59 fewer to 110 more) \| \| IBD Worsening (patient reported) – yes/no \| 54 (1 observational study) \| ⨁◯◯◯ Very low^b,c,d^ \| **OR 0.11** (0.01 to 1.07) \| Study population \| \| \| 211 per 1,000 \| **182 fewer per 1,000** (208 fewer to 11 more) \| \| Mortality (all cause) – yes/no \| 219 (6 observational studies) \| ⨁◯◯◯ Very low^a,b,c^ \| **OR 2.24** (0.22 to 22.96) \| Study population \| \| \| 0 per 1,000 \| **0 fewer per 1,000** (0 fewer to 0 fewer) \| \| Moderate \| \| \| 57 per 1,000 \| **45 fewer per 1,000** \| \| Reoperations (failure of primary bariatric procedure, IBD revisional surgery, or both) – yes/no \| 220 (6 observational studies) \| ⨁◯◯◯ Very low^a,b,c^ \| **OR 0.29** (0.07 to 1.15) \| Study population \| \| \| 123 per 1,000 \| **84 fewer per 1,000** (113 fewer to 16 more) \|  1. The included studies were deemed unclear to high risk of bias due to concerns over patients selection, comparability of the two groups, and variable follow up periods. 2. This outcome had a small sample size. The estimate of the effect is fragile. 3. The relative effect estimate for this outcome crosses the threshold of significance. 4. The included study was deemed unclear risk of bias on the basis of patient selection, comparability, and follow up period. | Moderate 8 |
| Undesirable Effects How substantial are the undesirable anticipated effects? | | |
| Judgement | Research evidence | Additional considerations |
| ○ Trivial ○ Small ○ Moderate ○ Large ○ Varies ○ Don't know |  |  |
| Certainty of evidence What is the overall certainty of the evidence of effects? | | |
| Judgement | Research evidence | Additional considerations |
| ○ Very low ○ Low ○ Moderate ○ High ○ No included studies |  |  |
| Values Is there important uncertainty about or variability in how much people value the main outcomes? | | |
| Judgement | Research evidence | Additional considerations |
| ○ Important uncertainty or variability ○**Possibly important uncertainty or variability** ○ Probably no important uncertainty or variability ○ No important uncertainty or variability |  | May depend on CD vs UC, how well-controlled the disease is at baseline. |
| Balance of effects Does the balance between desirable and undesirable effects favor the intervention or the comparison? | | |
| Judgement | Research evidence | Additional considerations |
| ○ Favors the comparison ○ Probably favors the comparison ○ Does not favor either the intervention or the comparison ○ Probably favors the intervention ○ **Favors the intervention** ○ Varies ○ Don't know |  |  |
| Resources required How large are the resource requirements (costs)?" | | |
| Judgement | Research evidence | Additional considerations |
| ○ Large costs ○ Moderate costs ○ Negligible costs and savings ○ Moderate savings ○ Large savings ○ Varies ○ Don't know |  |  |
| Certainty of evidence of required resources What is the certainty of the evidence of resource requirements (costs)? | | |
| Judgement | Research evidence | Additional considerations |
| ○ Very low ○ Low ○ Moderate ○ High ○ No included studies |  |  |
| Cost effectiveness Does the cost-effectiveness of the intervention favor the intervention or the comparison? | | |
| Judgement | Research evidence | Additional considerations |
| ○ Favors the comparison ○ Probably favors the comparison ○ Does not favor either the intervention or the comparison ○ Probably favors the intervention ○ Favors the intervention ○ Varies ○ No included studies |  |  |
| Equity What would be the impact on health equity? | | |
| Judgement | Research evidence | Additional considerations |
| ○ Reduced ○ Probably reduced ○ Probably no impact ○ Probably increased ○ Increased ○ Varies ○ Don't know |  |  |
| Acceptability Is the intervention acceptable to key stakeholders? | | |
| Judgement | Research evidence | Additional considerations |
| ○ No ○ Probably no ○ **Probably yes** ○ Yes ○ Varies ○ Don't know |  | quiescent IBD, high BMI, GERD… may be a preference for bypass |
| Feasibility Is the intervention feasible to implement? | | |
| Judgement | Research evidence | Additional considerations |
| ○ No ○ Probably no ○ Probably yes ○ **Yes** ○ Varies ○ Don't know |  |  |

# Summary of judgements

|  | **Judgement** | | | | | | |
| --- | --- | --- | --- | --- | --- | --- | --- |
| **Problem** | **No** | Probably no | Probably yes | Yes |  | Varies | Don't know |
| **Desirable Effects** | Trivial | Small | **Moderate** | Large |  | Varies | Don't know |
| **Undesirable Effects** | Trivial | Small | Moderate | Large |  | Varies | Don't know |
| **Certainty of evidence** | **Very** **low** | Low | Moderate | High |  |  | No included studies |
| **Values** | Important uncertainty or variability | **Possibly important uncertainty or variability** | Probably no important uncertainty or variability | No important uncertainty or variability |  |  |  |
| **Balance of effects** | Favors the comparison | Probably favors the comparison | Does not favor either the intervention or the comparison | Probably favors the intervention | **Favors the intervention** | Varies | Don't know |
| **Acceptability** | No | Probably no | **Probably yes** | Yes |  | Varies | Don't know |
| **Feasibility** | No | Probably no | Probably yes | **Yes** |  | Varies | Don't know |

# Type of recommendation

| Strong recommendation against the intervention | Conditional recommendation against the intervention | Conditional recommendation for either the intervention or the comparison | **Conditional recommendation for the intervention** | Strong recommendation for the intervention |
| --- | --- | --- | --- | --- |
| ○ | ○ | ○ | • | ○ |

# Conclusions

| Recommendation |
| --- |
| The panel suggests sleeve gastrectomy rather than bypass for obese patients with IBD. |
|  |

| Justification |
| --- |
|  |

| Subgroup considerations |
| --- |
| Patients with very high BMIs, GERD, and/or quiescent IBD may have preference for bypass rather than sleeve.  Esophageal dysmotility. DM.  Favor recommendation – Immunosuppression, pts who have undergone prior bowel resections |

| Implementation considerations |
| --- |
|  |

| Monitoring and evaluation |
| --- |
|  |

| Research priorities |
| --- |
| Same as discussed in KQ1  Disease remission and recurrence (IBD) |

# References Summary
